# Nursing Interventions That Promote Sleep in Preterm Newborns in the Neonatal Intensive Care Units: An Integrative Review

**DOI:** 10.3390/ijerph191710953

**Published:** 2022-09-02

**Authors:** Catarina Firmino, Marlene Rodrigues, Sofia Franco, Judicília Ferreira, Ana Rita Simões, Cidália Castro, Júlio Belo Fernandes

**Affiliations:** 1 Escola Superior de Saúde Egas Moniz, Caparica, 2829-511 Almada, Portugal; 2 Centro de Investigação Interdisciplinar Egas Moniz (CiiEM), 2829-511 Almada, Portugal; 3 Grupo de Patologia Médica, Nutrição e Exercício Clínico (PaMNEC), 2829-511 Almada, Portugal

**Keywords:** barriers, facilitators, person-centred care, rehabilitation, qualitative research

## Abstract

Sleep is a crucial factor for the psychological and physiological well-being of any human being. In Neonatal Intensive Care Units, preterm newborns’ sleep may be at risk due to medical and nursing care, environmental stimuli and manipulation. This review aims to identify the nurses’ interventions that promote sleep in preterm newborns in the Neonatal Intensive Care Units. An integrative review was conducted following Whittemore and Knafl’s methodology and the 2020 Preferred Reporting Items for Systematic Reviews and Meta-analyses (PRISMA) statement. The research was carried out on the electronic databases PubMed, Cochrane Central Register of Controlled Trials, Cochrane Database of Systematic Reviews, and ScienceDirect, with a timeframe from 2010 to 2021. A total of 359 articles were initially identified. After selection and analysis, five studies were included in the sample. Interventions by nursing staff that promote sleep in preterm newborns in the Neonatal Intensive Care Units fall within three categories: environmental management, relaxation techniques and therapeutic positioning. Nurses play a vital role in implementing interventions that promote preterm newborns’ sleep. They can positively affect preterm newborns’ sleep by controlling environmental stimuli and applying relaxation techniques and therapeutic positioning to their care practices.

## 1. Introduction

Worldwide, it is estimated that 5 million children are born preterm each year, and one million of these newborns will not survive the first year of life due to complications associated with prematurity [1].

In addition, complications associated with preterm delivery are the leading cause of death in children under five years of age [2]. It is estimated that three-quarters of these deaths can be prevented using the available interventions. These interventions should focus on using incubators, nasogastric tubes, and ventilators, in addition to implementing communication strategies aiming to reach at-risk populations and educating healthcare professionals and others in healthcare delivery systems to play key roles in reducing the risk of preterm birth. Furthermore, worldwide, the incidence of preterm births is rising with the result that prematurity is considered a major public health problem [3].

Preterm is defined as babies born alive before 37 weeks of pregnancy are completed [1]. Prematurity is the primary reason for hospitalization in neonatal intensive care units [4,5,6].

In addition to a lower birth weight, gestational age, and susceptibility to maturation and prematurity complications, preterm newborns in neonatal intensive care units are exposed to several stressors, including separation from the mother, frequent nursing or medical interventions, and excessive sound or light intensity [7,8,9].

In neonatal intensive care units, preterm newborns can be exposed to stressful events, such as painful clinical procedures, as many as 5 to 15 painful clinical procedures daily [10]. This stress may overwhelm the newborns and result in autonomic instability leading to adverse cardiac and respiratory changes and reduced levels of oxygen saturation. For example, pain can lead to significant changes in cardiac dynamics and loss of complexity of heart rate fluctuations. These changes include the altered fractal organization of heart rate variability that resemble some life-threatening conditions [11,12,13]. Stress has been associated with potentially long-lasting effects on newborns’ brain organization and neuroendocrine stress responses. Furthermore, current studies reported epigenetic changes in preterm newborns exposed to high-stress levels during the neonatal period [14,15]. Stress may also predispose preterm newborns to sleep disturbances caused by frequent interruptions or noise [13].

Sleep is the recurring physiologic state characterized by altered consciousness and is of great importance for optimal development [16].

Sleep is a critical issue in current neonatal intensive care unit practice for preterm newborns [17]. Fetuses and newborns spend most of their time sleeping. In the fetus, sleep and sleep cycles develop between 26 and 28 weeks of gestational age, and rapid eye movement sleep develops at around 28 to 30 weeks [18]. Even though quiet sleep and active sleep are present in newborns, *for reasons not known,* they do not establish a circadian rhythm for sleep. As a result, newborns are predisposed to have inefficient and easily interrupted sleep cycles [19].

In contrast, adults’ sleep occurs in two qualitatively different phases that display cyclicity: the active phase known as rapid eye movement sleep and the non-active phase called non-rapid eye movement sleep [16].

Newborns have different sleep patterns than infants and adults. The literature describes that, in newborns, sleep occurs in distinct phases, namely active sleep, quiet sleep, and indeterminate sleep [20]. In the active sleep phase, there is rapid eye movement as in older children and adults. This is the main sleep phase. In addition to rapid eye movement, it is also characterized by irregular breathing, sporadic motor movements, and a continuous electroencephalography pattern. In the quiet sleep phase, there is non-rapid eye movement, regular breathing patterns, an absence of motor movements, and a discontinuous electroencephalography pattern. Finally, in the indeterminate sleep phase, sleep characteristics cannot be clearly classified as active or quiet sleep [20,21]. 

The newborn sleep pattern organized into distinct phases allows normal neurodevelopmental outcomes. In addition, evidence shows that sleep cyclicity is key to the maintenance of brain plasticity, specifically the ability of the brain to reorganize its neural pathways in the face of environmental stimuli [22].

In preterm newborns, the lack of rest or sleep is a significant stressor and may harm the infant’s overall development [13]. Studies revealed that a longer sleep duration, regularity, and quality of sleep are associated with improved attention span, behavior, cognitive functioning, emotional regulation, and physical health in children [23]. Although the effects of sleep disturbance in preterm newborns are not known with certainty, sleep deprivation is associated with physiologic instability and less than optimal developmental outcomes. Current studies support that sleep plays a vital role in preterm newborns’ overall growth and development [17,24,25,26]. Consequently, it is considered a crucial action, similar to breathing and nutrition [25].

Preterm newborns receive multisensory stimulation in neonatal intensive care units, which can lead to difficulty establishing circadian rhythm [27]. In addition, newborns are exposed to continuous stimuli, leading to sleep-wake transition disruptions. A past study showed that newborns’ sleep is interrupted about 234 times in 24 h [28]. The main neonatal intensive care units’ environmental factors that disturb newborns’ sleep are sound and light intensity. When preterm newborns are admitted to the neonatal intensive care units, their optimal development may be at risk due to environmental stimuli, and medical and nursing care [25]. Therefore, special attention should be paid to preterm newborns’ sleep in the neonatal intensive care units. 

When caring for preterm newborns, nurses should be aware of sleep stages, the benefits of sleep, and its disturbing and inducing factors in planning and performing practices that cause minimal distress to preterm newborns. Consequently, developing nursing interventions that promote sleep for preterm newborns in neonatal intensive care units is an important area of research. This review aims to identify the nurses’ interventions that promote sleep in preterm newborns in neonatal intensive care units.

## 2. Methods

### 2.1. Design

The present integrative review was drawn based on the 2020 Preferred Reporting Items for Systematic Reviews and Meta-analyses (PRISMA) statement [29]. We used the methodological approach proposed by Whittemore and Knafl [30], involving five stages: (1) problem identification, (2) literature search, (3) data evaluation, (4) data analysis, and (5) presentation.

The question that guided this integrative review was defined in accordance with population, concept, and context (PCC) questions. What are nursing interventions (C) that promote sleep in the preterm newborn (P) hospitalized in neonatal intensive care units (C)?

### 2.2. Search Methods

The literature search was conducted using PubMed, Cochrane Central Register of Controlled Trials, Cochrane Database of Systematic Reviews, and ScienceDirect databases. The final search was performed on 7 October 2021.

Both DeCS and MeSH health sciences descriptors were combined with Boolean operators using the following search string:

(“Newborn” OR “Preterm”) AND (“Sleep”) AND (“Neonatal Intensive Care Unit”).

The selection criteria were: documents written in Portuguese and English, published between 2010 and 2021, which addressed or referred to the nursing interventions that promote sleep for preterm newborns (born at less than 37 weeks of gestation) receiving care in the NICU. All documents that did not meet the selection criteria were excluded from the review.

### 2.3. Study Selection

To increase consistency, the search, selection, and extraction of data were carried out independently by two researchers. After duplicate elimination, researchers proceeded with a selection process that enrolled three phases. In the first phase, researchers screened the titles, followed by abstract analysis. Finally, researchers obtained the full text of relevant documents and read them thoroughly. This process allowed verifying the relevance and appropriateness of the selected documents according to the inclusion and exclusion criteria and the research question. If there was disagreement, a third reviewer made the final decision.

### 2.4. Search Outcomes

**Figure 1:**
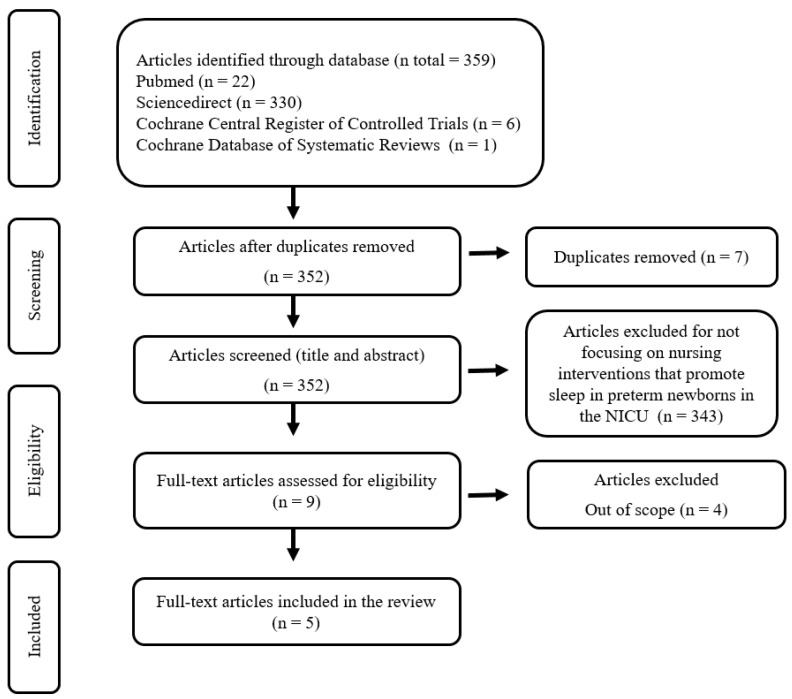
PRISMA flow diagram for study selection.

The initial database search identified 359 articles. After duplicate articles were removed, 352 titles and abstracts were reviewed, of which 9 were considered suitable for a full-text review. At the end of the screening process, five studies met the eligibility criteria and were included in this review. The flow chart describing the screening process is presented in Figure 1.

### 2.5. Quality Appraisal

Researchers defined the quality of the selected documents using the Joanna Briggs Institute levels of evidence and grading, ranging from 1c to 4b.

The Joanna Briggs Institute Critical Appraisal Checklist was applied to each study. 

The checklist aims to assess the study’s research design and the validity of its results using a sequence of appraisal questions with four possible answers (“yes”, “no”, “unclear”, or “not applicable”).

For scores below 49%, the study should be considered as having a high risk of bias, between 50% and 69% a moderate risk of bias, and more than 70% a low risk of bias.

The methodological rigor of the five studies selected by researchers ranged from 82% to 100%, which was considered a low risk of bias.

### 2.6. Data Extraction and Synthesis

A data extraction form was developed to guide the data extraction.

This instrument allowed the extraction of the following data: authors, publication year, study title, study design, aim, and findings. All data items extracted were cross-checked. 

## 3. Results

This integrative literature review allowed the identification of five articles that focus on sleep promotion in preterm newborns in the Neonatal Intensive Care Units. Out of the five studies, there were two studies conducted in the United States [13,31], one in Greece [32], one in Turkey [33], and another in Brazil [34]. Research studies were primarily involved randomized controlled trials [32,33], one randomized crossover pilot study [13], one systematic review of reviews [34], and one cross-sectional study [31].

A summary of the included articles with an overview of their key characteristics is provided in Table 1.

Data analysis revealed several nursing interventions that promote sleep in preterm newborns. Using an inductive analysis process, we grouped the different interventions into three categories based on the differences and similarities found between them. Each category is detailed below.

### 3.1. Category 1: Environmental Management

A study developed by Boutopoulou, Effrossine, Despoina, Konstatntinos, and Matziou [32] aimed to measure neonatal non-rapid eye movement sleep duration and how it was affected by the implementation of improved nursing conditions verified that, by reducing sound or light intensity, the duration of non-rapid eye movement sleep increased significantly. In this study, researchers applied specific earplugs (Minimuffs Neonatal Noise attenuators, Natus) to reduce the sound intensity and minimize ambient auditory stimuli reaching neonates’ ears. This intervention allowed to reduce the perceived volume by about 30 dB. For the reduction in light intensity, researchers used incubator covers.

Researchers concluded that providing low noise and light levels within neonatal intensive care units improves the structural organization of sleep with more prolonged non-rapid eye movement periods.

### 3.2. Category 2: Relaxation Techniques

In the category relaxation techniques, pediatric massage emerged as an intervention that promotes sleep in newborns. Two articles address this intervention. First, the study conducted by Yates, Mitchell, Booth, Williams, Lowe, and Hall [13] aimed to determine whether massage therapy can be used as an adjunct intervention to induce sleep in infants born preterm. Researchers adapted the massage protocol originally published by Field et al. [35], which resulted in an overall massage time of approximately 10 min. 

Baby lotion (Johnson & Johnson) was used to assist with ease of skin-to-skin contact during moderate pressure massage. The infant was undressed to the diaper and covered with a blanket during massage to maintain warmth. Each infant received two repetitions of a series of defined strokes to five body areas. Massage occurred over 1 min intervals with the application of 12 strokes lasting approximately five seconds each for each of the body areas receiving massage. The following sequence was performed: (1) in the prone position, the infant was stroked from the top of the head to the neck and back to the top of the head and back to the neck; (2) from the neck across the shoulders; (3) from the upper back to the waist and back to the upper back; (4) from the thigh to the foot and back to the thigh on both legs; and (5) from the shoulder to the hand and back to the shoulder on both arms.

The review performed by de Britto Pereira, Mendes Abdala, Portella, Ghelman, and Schveitzer [34] analyzed 38 reviews that evaluated pediatrics’ massage as an intervention in several health outcomes. The outcomes were divided into four major groups: physical and metabolic effects; vitality, well-being, and quality of life; mental health; and management. This review showed the positive effects of massage in promoting quality of sleep.

Another intervention that promotes sleep in newborns that encompasses the category relaxation techniques is tub bathing.

In the study developed by Taşdemir and Efe [33], tub bathing emerged as an intervention with positive results in promoting sleep in the preterm newborn. Bathing was performed anywhere from 6 to 48 h post-birth, between 8 a.m. and 11 a.m., with minimal nutritional and other interventions provided to the infant during this period. 

Bathing was performed at room temperature measured at 25–26 °C and 40% humidity. The water level in the bath was set at approximately 9–12 cm or deep enough to cover the baby’s shoulders. The bathwater temperature was controlled using a water thermometer and set at 37–38 °C. A folded cloth towel was placed into the tub before bathing. During bathing, infants were spoken to softly, and their bodies were cleaned in a slow, rhythmical motion. The infant’s face was washed and dried before immersion. First, the lower part of the body was immersed in a tub before immersion up to the neck. The infant was held securely; the head and neck were supported on the researcher’s forearm, and the shoulder was grasped using the researcher’s thumb and finger. Cleaning was performed using a soft cloth and an infant skin cleaner. The front and back areas were cleaned without turning the infant. Bathing took approximately 3.64 ± 0.77 min. Then, the baby was safely removed from the water and wrapped in a clean, soft towel. The body was quickly dried with gentle movements, baby oil applied, and the nappy put on.

### 3.3. Category 3: Therapeutic Positioning

A study developed by Zarem, Crapnell, Tiltges, Madlinger, Reynolds, Lukas, and Pineda [31], aiming to determine perceptions about positioning for preterm infants in the neonatal intensive care units, identified that, in comparison to other positioning methods (i.e., Sleep Sack, Snuggle Up, nesting, boundaries, and swaddling), the Dandle Roo is the easiest to use and most beneficial. The Dandle Roo is a device made of stretchable cotton that provides containment, allowing the infant to move the extremities into extension, followed by recoil to flexion and midline orientation [31]. Although the participants recognized the benefits of this positioning method, they acknowledged that good positioning might be achieved in various ways. For example, the use of nesting and blankets can still facilitate positive results for the preterm infant.

## 4. Discussion

The current review provides a comprehensive understanding of nursing interventions that promote sleep in preterm newborns hospitalized in the neonatal intensive care units. A total of three categories of interventions (*environmental* management, relaxation techniques, and therapeutic positioning) were identified from five studies. The results show that nurses can implement different strategies to promote sleep in preterm newborns hospitalized in neonatal intensive care units.

Preterm newborns are exposed to various stimuli in neonatal intensive care units, causing frequent sleep–wake transition disruptions and leading to sleep disorganization. In addition, neonatal intensive care units are excessively sensory environments with high sound and light intensity levels. These two factors are the most common environmental factors that disturb neonatal sleep [36,37,38,39]. 

In most neonatal intensive care units, the light comes from artificial sources, such as examination lights, phototherapy lamps, and ambient space light, varying in intensity according to the unit’s needs during the 24 h day. The sound is mainly produced by healthcare workers and medical devices, such as monitors, respiratory equipment, and double-walled incubators. 

Several studies associate the light and sound intensity with autonomic nervous system disorders, increased heart rate and vasoconstriction, delay in obtaining complete enteral nutrition, and uncontrolled circadian cycle [40]. There is also evidence that newborns’ non-rapid eye movement sleep increases when sound or light intensity is reduced [32]. Therefore, improving these parameters of nursing practices may facilitate the newborns’ sleep duration and result in better neurodevelopmental outcomes [41].

Despite the positive conclusions drawn by Boutopoulou, Effrossine, Despoina, Konstatntinos, and Matziou [32] regarding the use of earplugs to promote newborns’ sleep, the authors considered that this method cannot be regarded as a practical clinical approach for noise reduction. Instead, the authors suggest alternative nursing practices such as lowering the tone of conversations and reducing the intensity of noises produced by alarms of monitoring devices. 

Concerning incubator covers, this is already a method instituted by many neonatal intensive care units to minimize the intensity of light affecting newborns [42]. 

Among the relaxation techniques to help to promote sleep, pediatric massage is an inexpensive intervention that should be incorporated into nursing practices.

For long, the introduction of massage therapy into nursing practices has been delayed due to fear of overstimulating the infant. However, evidence supports its safety and shows that the significant benefits outweigh the minimal risks [34]. In the study conducted by Yates, Mitchell, Booth, Williams, Lowe, and Hall [13], although newborns did not demonstrate induction of sleep immediately after the massage, their response of increased wakefulness may be enlightened by evidence that massage therapy enhances the electrical activity and brain maturation in preterm newborns [43,44]. The review performed by de Britto Pereira, Mendes Abdala, Portella, Ghelman, and Schveitzer [34] revealed the benefits of implementing massage as a sleep-promoting strategy in preterm newborns. In addition, other benefits of massage therapy include the stabilization of the autonomic nervous system, promotion of growth and development, and shorter hospital stays [45,46,47].

Current evidence shows that massage therapy is a low-cost, safe practice associated with multiple benefits for preterm newborns and, therefore, should be incorporated into nurses’ practice.

Another relaxation technique identified in this review was tub bathing. The study developed by Taşdemir and Efe [33] suggests that tub bathing can be effective in reducing infant crying and helping them to sleep. In addition, when compared with sponge bathing, tub bathing had a more significant stress reduction effect in preterm newborns.

Bathing can be an extremely stressful agent for preterm newborns, which may lead to some behaviors, such as agitation, crying, and hiccoughing [48]. However, in Taşdemir and Efe’s [33] study, tub bathing positively affected the newborns’ comfort and helped them to maintain their regular heart rate. Consequently, tub bathing is a stress reduction intervention that promotes sleep in preterm newborns in neonatal intensive care units. The evidence provided by this study supports the need to emphasize the nurses’ role in the care provided to preterm newborns and the integration of tub bathing in nurses’ practice. Furthermore, the results of this study indicate that tub bathing helps to prevent thermodynamic instability, stress, and impairments in physiological parameters. For this reason, it is an important method to be promoted to obtain better health outcomes, specifically, sleep promotion. 

Finally, the use of positioning aids was identified as a nurse intervention that promotes sleep in preterm newborns. The last pregnancy trimester encourages the development of physiologic flexion and midline orientation [49,50], preparing the fetus for later function, supporting neurodevelopment, and promoting self-soothing [51]. By being born preterm, newborns are deprived of this critical experience, frequently resulting in low muscle tone and strength that cause them to maintain their bodies in extended positions [52,53]. This position may affect the newborns’ development, inhibit self-regulation [54], and interfere with their ability to interact and attach to their caregivers [31]. 

Neonatal intensive care unit nurses are responsible for multiple aspects of care. Therapeutic positioning is one of these aspects of care that can have critical developmental effects on preterm newborns. To minimize the sequelae of prematurity, nurses try to encourage adequate positioning using various methods. The study developed by Zarem, Crapnell, Tiltges, Madlinger, Reynolds, Lukas, and Pineda [31] identified that the Dandle Roo was considered the most accessible positioning aid and the most beneficial one. However, other aids, such as the sleep sack, snuggle up, nesting, boundaries, and swaddling, are also effective in promoting the newborn’s adequate positioning and sleep.

Nurses must be aware of the importance of sleep for preterm newborns as a necessary neurodevelopmental process and also be acquainted with interventions that promote sleep in this population. The findings from this review allowed us to understand that nurse practice has a predominant effect on preterm newborn development. Several interventions were identified to promote sleep in preterm newborns in the neonatal intensive care units. In addition, it is also essential to educate and include parents to promote appropriate care to meet the newborns after discharge. 

This review identifies several studies that report sufficient detail of its interventions permitting replication. This state of play allows nurses to implement those interventions in their daily practice. Nonetheless, this research has several limitations. First, limiting the search to four databases and imposing time limits may have excluded some relevant studies. Second, we have to consider the exclusion of the literature written in languages other than English and Portuguese. Third, the low number of studies included in the review. While there has been an increasing body of evidence focusing on preterm newborns, this review shows a gap regarding the nursing interventions for promoting sleep in preterm newborns as only five studies were identified. Based on the possible multiple benefits of sleep promotion care practices for preterm newborns and the scarcity of studies focusing on this subject, further research is needed.

## 5. Conclusions

Sleep is generally considered vital for brain development and growth during the neonatal period. In addition, identifying and implementing appropriate nursing care practices that ensure physiologic stability, growth and development, rest, sleep, and the ability to cope with procedures and other numerous stressors are believed to positively affect the newborns’ sleep organization. Therefore, sleep promotion is essential for preterm newborns’ optimal development.

The findings of this review will contribute to the advancement in nursing care practice addressing a critical issue of improving care of preterm newborns in the NICU impacting their early development. By managing the environment by reducing sound and light intensity, nurses ensure that the ambient stimuli that reach newborns are minimized and, therefore, promote their sleep duration and result in better neurodevelopmental outcomes. Better sleep outcomes can also be achieved by introducing relaxation techniques, such as pediatric massage or tub bathing in nursing practice. In addition, therapeutic positioning, with or without positioning aids, can also positively affect preterm newborns’ sleep.

## Figures and Tables

**Table 1:** Data extraction and synthesis.

Author/Year/Title/Country	Study Design	Aim	Population	Interventions
Zarem, Crapnell, Tiltges, Madlinger, Reynolds, Lukas and Pineda [31] (2013) Neonatal nurses’ and therapists’ perceptions of positioning for preterm infants in the neonatal intensive care unit USA	Cross-sectional study	To identify neonatal nurses’ and neonatal therapists’ perceptions about different methods of positioning used in the study’s neonatal intensive care units.	Neonatal nurses and speech, physical, and occupational therapists.	Therapeutic positioning
Yates, Mitchell, Booth, Williams, Lowe and Hall [13] (2014) The effects of massage therapy to induce sleep in infants born preterm USA	Randomized crossover pilot study	To determine whether massage therapy can be used as an adjunct intervention to induce sleep in infants born preterm.	30 preterm newborns. Gestational age of 32–48 weeks.	Massage therapy
Boutopoulou, Effrossine, Despoina, Konstatntinos and Matziou [32] (2016) Effects of neonatal intensive care unit nursing conditions in neonatal non-rapid eye movement sleep Greece	Randomized controlled trial	To investigate the relation between noise and light levels in the neonatal intensive care units environment and non-rapid eye movement sleep duration	46 preterm newborns. Gestational age mean ± SD = 35.13 ± 3.12 weeks.	Application of earplugs (Minimuffs Neonatal Noise attenuators, Natus) to reduce the sound intensity and minimize ambient auditory stimuli reaching neonates’ ears. Application of incubator covers to reduce light intensity.
Taşdemir and Efe [33] (2019) The effect of tub bathing and sponge bathing on neonatal comfort and physiological parameters in late preterm infants: A randomized controlled trial Turkey	Randomized controlled trial	To examine the effectiveness of tub bathing and sponge bathing methods on the physiological parameters (i.e., heart rate, respiration rate, oxygen saturation, and body temperature) and comfort of late preterm infants.	120 preterm newborns. Group 1—gestational age mean ± SD = 35.28 ± 1.83 3 weeks. Group—gestational age mean ± SD = 35.31 ± 1.01 weeks.	Tub bathing
de Britto Pereira, Mendes Abdala, Portella, Ghelman and Schveitzer [34] (2021) Pediatrics massage evidence map Brazil	Systematic review of reviews	To describe different pediatrics’ massage interventions and report-related health outcomes.	38 reviews	Massage therapy

## Data Availability

The data presented in this study are available upon request from J.B.F.
